# Thyroidectomy for hyperthyroidism before the euthyroid state: is it safe?

**DOI:** 10.1186/s12893-025-03195-y

**Published:** 2025-10-03

**Authors:** Muhammer Ergenç, Sena Altunsu, Fatma Nazlı Zorlu, Ahmet Akmercan, M. Ümit Uğurlu

**Affiliations:** 1 Breast and Endocrine Surgery Unit, Department of General Surgery, Marmara University School of Medicine, Istanbul, Turkey; 2 Department of General Surgery, Marmara University School of Medicine, Istanbul, Turkey; 3 Department of General Surgery, Marmara University Pendik Training and Research Hospital, Istanbul, Turkey

**Keywords:** Graves’ disease, Thyroid storm, Thyroidectomy, Thyrotoxicosis, Hyperthyroidism, Surgery

## Abstract

**Background:**

Guidelines generally recommend achieving a biochemically euthyroid state before thyroidectomy in patients with an indication for surgery to reduce the risk of thyroid storm. However, in real-world settings, this may not always be feasible. This study aimed to compare perioperative outcomes between biochemically controlled (normal fT3 and fT4) and uncontrolled (elevated fT3 and/or fT4) hyperthyroid patients undergoing thyroidectomy.

**Methods:**

We retrospectively analyzed patients who underwent thyroidectomy for hyperthyroidism at our institution between September 2020 and 2024. The demographic, perioperative, and postoperative data were collected. Patients with preoperative fT3 and/or fT4 levels above the institutional reference range were classified as uncontrolled, whereas those with both fT3 and fT4 levels within the reference range were considered controlled. The outcomes were compared between the controlled and uncontrolled groups.

**Results:**

A total of 110 patients were included: 92 (83.6%) in the controlled group and 18 (16.4%) in the uncontrolled group. Patients in the uncontrolled group were significantly younger (median age 33.5 vs. 49 years, *p* = 0.015). Graves’ disease was more prevalent among uncontrolled patients (83.3% vs. 45.7%, *p* = 0.013). The use of Lugol’s iodine (27.8% vs. 1.1%, *p* < 0.001) and steroids (38.9% vs. 6.5%, *p* < 0.001) was significantly higher in the uncontrolled group than in the control group. Operative times and complication rates—including transient/permanent hypocalcemia, recurrent laryngeal nerve palsy, and neck hematoma—did not significantly differ between the groups.

**Conclusions:**

Despite the presence of biochemical hyperthyroidism, no thyroid storm occurred in our cohort, and complication rates were comparable between groups. These findings suggest that thyroidectomy may be performed in selected patients without full biochemical control, particularly in urgent situations or in high-volume centers with experienced surgical teams. However, biochemical euthyroidism remains the standard of care, and our results should be interpreted cautiously given the small sample size and single-center setting.

## Introduction

Overt hyperthyroidism affects approximately 0.2–1.3% of individuals in iodine-sufficient regions, representing a notable public health concern. Graves’ disease accounts for 50–80% of all cases, followed by toxic multinodular goiter and solitary toxic adenoma. Treatment options include antithyroid drugs, radioactive iodine (RAI), and surgery, with distinct indications, risks, and benefits. The primary goals of therapy are symptom control and prevention of thyrotoxicosis-related complications, including thyroid storm [1, 2].

Antithyroid medications such as methimazole and propylthiouracil can cause significant side effects, including hepatotoxicity, agranulocytosis, and teratogenicity. RAI is contraindicated in patients with thyroid eye disease and in pregnant women. Moreover, both medical and RAI therapies may take several weeks to months to restore euthyroidism and sometimes fail, requiring retreatment. In contrast, when performed by experienced endocrine surgeons, thyroidectomy provides a definitive cure and is associated with low complication rates. Nonetheless, the procedure carries risks such as recurrent laryngeal nerve injury, hypoparathyroidism, hemorrhage, and surgical site infection [3–7].

Thyroid storm is an extreme manifestation of thyrotoxicosis that can be triggered by surgery or other physiological stressors. It is characterized by multiorgan dysfunction and has a mortality rate of up to 30%. Diagnostic criteria include suppressed thyroid-stimulating hormone (TSH) (< 0.01 mU/L), elevated free triiodothyronine (fT3) and/or free thyroxine (fT4), the presence of thyrotropin receptor antibodies (TRAb) (which is associated with Graves’ disease) along with clinical signs and symptoms of end-organ damage. The Burch-Wartofsky Point Scale (BWPS) and the Japanese Thyroid Association (JTA) scale are widely used for diagnostic assessment. Treatment requires prompt, aggressive medical management to reverse thyrotoxicosis and prevent organ failure [8–10].

Current guidelines, including those by the American Thyroid Association (ATA) and the American Association of Endocrine Surgeons (AAES), recommend achieving euthyroidism before thyroidectomy to reduce the risk of thyroid storm. These recommendations are largely based on limited evidence. The rationale includes concerns that surgical manipulation of the thyroid gland may provoke hormone release and that general anesthesia may precipitate decompensation in thyrotoxic patients [3–6, 11, 12].

However, achieving a euthyroid state preoperatively is not always feasible. Factors such as drug intolerance, side effects, poor treatment adherence, resistance to therapy, or the need for urgent surgical intervention may preclude preoperative normalization of hormone levels. In clinical practice, some patients undergo surgery while being biochemically hyperthyroid despite medical optimization attempts [3–6].

This study aimed to evaluate the safety of thyroidectomy in patients with hyperthyroidism who had not achieved a preoperative euthyroid state by comparing perioperative outcomes between biochemically controlled (normal fT3 and fT4) and uncontrolled (elevated fT3 and/or fT4) groups.

## Materials and methods

This retrospective study included patients who underwent thyroidectomy for hyperthyroidism at Marmara University Hospital between September 2020 and September 2024. All patients were followed postoperatively in the outpatient clinic for general surgery. The study was approved by the Clinical Research Ethics Committee of Marmara University School of Medicine (Approval No: 2024.1079). As this was a retrospective study, the ethics committee waived the requirement for informed consent.

Patients diagnosed with hyperthyroidism by endocrine and general surgery teams and treated surgically were eligible for inclusion. Patients who underwent surgery for non-hyperthyroid indications or had incomplete diagnostic or follow-up data were excluded.

The following variables were collected from patient records: age, sex, body mass index (BMI), American Society of Anesthesiologists (ASA) score, smoking status, preoperative symptoms, comorbidities (including diabetes mellitus, hypertension, ischemic heart disease, dyslipidemia, chronic obstructive pulmonary disease, chronic kidney disease, cerebrovascular disease, and others), etiology of hyperthyroidism (Graves’ disease, toxic multinodular goiter, single adenoma), use and duration of antithyroid drugs (ATDs), preoperative medications (methimazole, propylthiouracil, beta-blockers, Lugol’s iodine, steroids), preoperative thyroid hormone levels (TSH, fT3, fT4), thyroid hormone status (euthyroid, subclinical hyperthyroid, hyperthyroid), indication for surgery (e.g., drug intolerance, allergic reaction, refractory symptoms, suspected malignancy, risk of thyroid storm), urgency of surgery (elective vs. emergency), type of procedure (total or hemithyroidectomy), operative time, postoperative complications (transient and permanent hypocalcemia, recurrent laryngeal nerve palsy, neck hematoma, emergency department visits within 30 days), and final histopathology results.

Marmara University Hospital is a high-volume center for endocrine surgery, where the majority of procedures were performed by experienced, high-volume endocrine surgeons [13]. Intraoperative nerve monitoring was utilized in nearly all cases. Institutional reference ranges were as follows: TSH (0.38–5.33 mIU/L), free T3 (2.13–4.50 ng/L), free T4 (0.58–1.38 ng/dL), PTH (15–65 ng/L), and serum calcium (8.8–10.6 mg/dL).

The diagnosis of hyperthyroidism was confirmed based on clinical evaluation, thyroid function tests, TRAb levels, thyroid scintigraphy, and ultrasonography [2]. Hyperthyroidism was defined as suppressed TSH with elevated fT3 and/or fT4 levels, whereas subclinical hyperthyroidism was defined as suppressed TSH with normal fT3 and fT4 levels [6]. Hypocalcemia was defined as a serum calcium level below the lower institutional reference limit. It was classified as transient if calcium supplementation was required for less than six months after surgery and as permanent if supplementation was required beyond six months [14]. Recurrent laryngeal nerve (RLN) palsy was considered transient if vocal cord mobility recovered within six months postoperatively and permanent if recovery did not occur [15]. The BWPS was used to assess suspected thyroid storm [8, 9].

The vast majority of patients with hyperthyroidism referred for surgical treatment were treated by an endocrinologist prior to surgery. Patients with poorly controlled hyperthyroidism and/or those requiring rapid preoperative optimization were treated with a combination of antithyroid drugs (ATDs), Lugol’s iodine, cholestyramine, beta-blockers, and corticosteroids to achieve rapid control of thyrotoxicosis. Signs of thyroid storm were closely monitored intraoperatively. All patients underwent routine monitoring, including electrocardiography, noninvasive blood pressure measurement, pulse oximetry, and temperature assessment. In addition, patient-specific monitoring and treatment strategies—such as invasive arterial blood pressure monitoring, intravenous corticosteroids, and beta-blockers—were implemented on an individualized basis.

Patients were categorized as uncontrolled if their preoperative fT3 and/or fT4 levels exceeded the institutional reference ranges on the day before surgery, regardless of whether they had received adjunctive therapies such as Lugol’s iodine, corticosteroids, or beta-blockers to improve perioperative stability. Patients with both hormone values within the normal ranges were considered controlled [4]. Thus, classification was based on biochemical criteria, while acknowledging that some patients in the uncontrolled group had achieved partial clinical stability through individualized perioperative management. In most cases, uncontrolled patients had either urgent surgical indications (e.g., thyroid storm, medication allergy, refractory thyrotoxic symptoms) or failed to achieve biochemical control despite appropriate medical therapy due to resistance, intolerance, or nonadherence. The perioperative outcomes were then compared between the two groups.

### Statistical analysis

All analyses were performed using IBM SPSS Statistics for Mac, Version 24. Continuous variables were expressed as mean ± standard deviation (SD) for normally distributed data or as median (interquartile range [IQR]) for non-normally distributed data. The categorical variables were compared using the chi-square test. Student’s t-test was used for normally distributed continuous data, and the Mann–Whitney U test was used for nonparametric comparisons. A two-tailed p-value < 0.05 was considered statistically significant, with a 95% confidence interval.

## Results

### Patient characteristics

A total of 110 patients were included in the analysis. Of these, 92 (83.6%) were categorized as controlled and 18 (16.4%) as uncontrolled based on their preoperative thyroid hormone levels. The median age of the patients in the uncontrolled group was significantly lower than that of the controlled group (33.5 vs. 49 years, *p* = 0.015). No statistically significant differences were observed between the groups in terms of sex distribution (*p* = 0.882), body mass index (*p* = 0.182), smoking status (*p* = 0.568), ASA physical status classification (*p* = 0.460), or the presence of comorbid conditions **(**Table 1**)**.

**Table 1:** Patient demographic and clinical characteristics

Variables	All patients	Controlled	Uncontrolled	*p*-value
	*N* = 110	*n* = 92	*n* = 18	
Age (years) (median, IQR)	49 (22)	49 (18)	33.5 (18)	0.015
Sex, n (%)				0.882
Female	81 (73.6)	68 (73.9)	13 (72.2)	
Male	29 (26.4)	24 (26.1)	5 (27.8)	
BMI (kg/m^2^), mean ± SD	27.6 ± 5.6	27.9 ± 5.4	25.7 ± 6.4	0.182
Smoking status				0.568
Never smoked	72 (65.5)	62 (67.4)	10 (55.6)	
Current smoker	26 (23.6)	21 (22.8)	5 (27.8)	
Ex-smoker (> 6 weeks)	12 (10.9)	9 (9.8)	3 (16.7)	
ASA Score, n (%)				0.460
1	12 (10.9)	11 (12)	1 (5.6)	
2	94 (85.5)	77 (83.7)	17 (94.4)	
3	4 (3.6)	4 (4.3)	0	
Comorbidities, n (%)				
Diabetes mellitus	13 (11.8)	10 (10.9)	3 (16.7)	0.444
Hypertension	32 (29.1)	29 (31.5)	3 (16.7)	0.204
Ischemic heart disease	8 (7.3)	6 (6.5)	2 (11.1)	0.615
Dyslipidaemia	3 (2.7)	2 (2.2)	1 (5.6)	0.418
Chronic obstructive pulmonary disease	1 (0.9)	1 (1.1)	0	0.836
Chronic kidney disease	1 (0.9)	1 (1.1)	0	0.836
Cerebrovascular disease	3 (2.7)	2 (2.2)	1 (5.6)	0.418
Others	21 (19.1)	19 (20.7)	2 (11.1)	0.516
Etiology of hyperthyroidism, n (%)				0.013
Graves’ disease	57 (51.8)	42 (45.7)	15 (83.3)	
Toxic multinodular goiter	43 (39.1)	41 (44.6)	2 (11.1)	
Single adenoma	10 (9.1)	9 (9.8)	1 (5.6)	
Preoperative ATDs, n (%)				0.639
Absent	9 (8.2)	7 (7.6)	2 (11.1)	
Present	101 (91.8)	85 (92.4)	16 (88.9)	
Length of time on ATDs (months) (median, IQR)	18 (33)	21.5 (33)	7.5 (22)	0.073
Methimazole, n (%)				0.359
Absent	13 (11.8)	10 (10.9)	3 (16.7)	
Present	97 (88.2)	82 (89.1)	15 (83.3)	
Propylthiouracil, n (%)				0.140
Absent	92 (83.6)	79 (85.9)	13 (72.2)	
Present	18 (16.4)	13 (14.1)	5 (27.8)	
Beta-Blockers, n (%)				0.019
Absent	64 (58.2)	58 (63)	6 (33.3)	
Present	46 (41.8)	34 (37)	12 (66.7)	
Lugol’s iodine, n (%)				< 0.001
Absent	104 (94.5)	91 (98.9)	13 (72.2)	
Present	6 (5.5)	1 (1.1)	5 (27.8)	
Steroids, n (%)				< 0.001
Absent	97 (88.2)	86 (93.5)	11 (61.1)	
Present	13 (11.8)	6 (6.5)	7 (38.9)	
Patient’s thyroid hormone status, n (%)				< 0.001
Euthyroid	58 (52.7)	58 (63)	0	
Subclinical hyperthyroid	28 (25.5)	28 (30.5)	0	
Hyperthyroid	5 (4.5)	0	5 (27.8)	
TSH < 0.01	19 (17.3)	6 (6.5)	13 (72.2)	
Preoperative TSH (mIU/L) (0.38–5.33) (median, IQR)	0.63 (1.78)	0.85 (1.99)	0.005 (0.005)	< 0.001
Preoperative Free T4 (ng/dL) (0.58–1.38) (median, IQR)	1.12 (0.47)	1.06 (0.37)	1.88 (1.57)	< 0.001
Preoperative Free T3 (ng/L) (2.13–4.50) (median, IQR)	3.2 (0.88)	3.14 (0.67)	4.05 (2.03)	0.001

*IQR* interquartile range, *SD* standard deviation, *BMI* body mass index, *ASA* American Society of Anesthesiologists physical status, *ATDs* antithyroid drugs

### Preoperative clinical and biochemical findings

Overall, Graves’ disease was the most common etiology of hyperthyroidism and was significantly more prevalent in the uncontrolled group than in the controlled group (83.3% vs. 45.7%, *p* = 0.013). Conversely, toxic multinodular goiter was more frequent in the controlled group (44.6% vs. 11.1%). Although the rate of antithyroid drug (ATD) use was similar between the groups (*p* = 0.639), the duration of ATD therapy tended to be shorter in the uncontrolled group (7.5 vs. 21.5 months), although this did not reach statistical significance (*p* = 0.073). The use of Lugol’s iodine (27.8% vs. 1.1%, *p* < 0.001) and corticosteroids (38.9% vs. 6.5%, *p* < 0.001) was significantly higher among uncontrolled patients.

Biochemical profiles differed significantly: 72.2% of patients in the uncontrolled group had suppressed TSH levels < 0.01 mIU/L, compared to 6.5% in the controlled group (*p* < 0.001). Median preoperative free T4 (1.88 vs. 1.06 ng/dL, *p* < 0.001) and free T3 (4.05 vs. 3.14 ng/L, *p* = 0.001) levels were also significantly higher in the uncontrolled group **(**Table 1**)**.

### Operative and postoperative outcomes

The most common indication for surgery was refractory symptoms (63.6%), followed by suspicion of malignancy(20.9%). Emergency surgeries were significantly more frequent in the uncontrolled group (27.8% vs. 1.1%, *p* < 0.001). Among the uncontrolled patients, five underwent emergency thyroidectomy. Three patients were diagnosed with thyroid storm prior to surgery, one required surgery due to antithyroid medication allergy, and one due to refractory thyrotoxic symptoms unresponsive to medical treatment. All cases were surgically managed following multidisciplinary consensus in response to clinical deterioration **(**Table 2**)**.

**Table 2:** Comparison of perioperative characteristics and outcomes between the two groups

Variables	All patients	Controlled	Uncontrolled	*p*-value
	*N* = 110	*n* = 92	*n* = 18	
Reason for surgery, *n* (%)				< 0.001
Allergy to medications	4 (3.6)	2 (2.2)	2 (11.1)	
Intolerance to medications	8 (7.3)	4 (4.3)	4 (22.2)	
Refractory symptoms	70 (63.6)	62 (67.4)	8 (44.4)	
Suspicion of malignancy	23 (20.9)	22 (23.9)	1 (5.6)	
Thyroid storm	5 (4.5)	2 (2.2)	3 (16.7)	
Urgency of surgery, n (%)				< 0.001
Elective	104 (94.5)	91 (98.9)	13 (72.2)	
Emergency	6 (5.5)	1 (1.1)	5 (27.8)	
Type of procedure, n (%)				0.275
Total thyroidectomy	103 (93.6)	85 (92.4)	18 (100)	
Hemithyroidectomy	7 (6.4)	7 (7.6)	0	
Operative time, n (%)				0.433
< 1 h	17 (15.5)	16 (17.4)	1 (5.6)	
1–2 h	69 (62.7)	56 (60.9)	13 (72.2)	
>2 h	24 (21.8)	20 (21.7)	4 (22.2)	
Postoperative complications				0.606
Absent	73 (66.4)	62 (67.4)	11 (61.1)	
Present	37 (33.6)	30 (32.6)	7 (38.9)	
Transient hypocalcemia				0.672
Absent	72 (65.5)	61 (66.3)	11 (61.1)	
Present	38 (34.5)	31 (33.7)	7 (38.9)	
Permanent hypocalcemia				0.615
Absent	102 (92.7)	86 (93.5)	16 (88.9)	
Present	8 (7.3)	6 (6.5)	2 (11.1)	
Transient recurrent nerve palsy				0.516
Absent	106 (96.4)	89 (96.7)	17 (94.4)	
Present	4 (3.6)	3 (3.3)	1 (5.6)	
Permanent recurrent nerve palsy				0.698
Absent	108 (98.2)	90 (97.8)	18 (100)	
Present	2 (1.8)	2 (2.2)	0	
Neck hematoma				0.667
Absent	104 (94.5)	87 (94.6)	17 (94.4)	
Present	6 (5.5)	5 (5.4)	1 (5.6)	
Postoperative hematoma requiring surgical evacuation				0.582
Absent	107 (97.3)	89 (96.7)	18 (100)	
Present	3 (2.7)	3 (3.3)	0	
Emergency department visit within 30 days				0.010
Absent	92 (83.6)	81 (88)	11 (61.1)	
Present	18 (16.4)	11 (12)	7 (38.9)	
Reason for visiting the emergency department				0.040
Absent	92 (83.6)	81 (88)	11 (61.1)	
Normal postoperative swelling and/or pain	10 (9.1)	6 (6.5)	4 (22.2)	
Numbness/tingling (normal calcium)	2 (1.8)	1 (1.1)	1 (5.6)	
Numbness/tingling (low calcium)	6 (5.5)	4 (4.3)	2 (11.1)	
Pathology				0.474
Benign	93 (84.5)	79 (85.9)	14 (77.8)	
Malignancy	17 (15.5)	13 (14.1)	4 (22.2)	

Total thyroidectomy was performed in 93.6% of all cases, with no significant difference in the extent of surgery between the two groups (*p* = 0.275). The mean operative time did not differ significantly (*p* = 0.433) **(**Table 2**)**.

Overall, postoperative complications occurred in 33.6% of the patients. The rates of specific complications—including transient hypocalcemia (*p* = 0.672), permanent hypocalcemia (*p* = 0.615), transient recurrent laryngeal nerve palsy (*p* = 0.516), permanent recurrent laryngeal nerve palsy (*p* = 0.698), and neck hematoma (*p* = 0.667)—did not significantly differ between the groups. However, emergency department visits within 30 days postoperatively were significantly more common in the uncontrolled group (38.9% vs. 12%, *p* = 0.010). These visits were primarily due to postoperative neck swelling, pain, or hypocalcemia-related symptoms (*p* = 0.040) **(**Table 2**)**.

### Subgroup analysis in patients with Graves’ disease

To further address the potential confounding effect of underlying etiology, we conducted a subgroup analysis limited to patients diagnosed with Graves’ disease (*n* = 57), comparing those with controlled (*n* = 42) and uncontrolled (*n* = 15) thyroid function. As expected, uncontrolled patients exhibited significantly higher preoperative thyroid hormone levels and more frequent use of adjunctive medications, such as Lugol’s iodine and corticosteroids. Despite these differences, the operative duration, perioperative complication rates, and early postoperative outcomes did not differ significantly between the groups. Given the small sample size and limited statistical power of this subgroup, the results should be interpreted with caution and were excluded from the main tables.

### Histopathological findings

The final pathology revealed benign disease in the majority of patients (84.5%), with no significant differences between the groups. Malignancy was detected in 22.2% and 14.1% of patients in the uncontrolled and controlled groups, respectively (*p* = 0.474) (Table 2).

## Discussion

The results of the present study indicate that thyroidectomy was technically feasible in patients who remained biochemically hyperthyroid at the time of surgery. Importantly, no thyroid storm was observed. However, given the rarity of this complication and the limited number of patients in the uncontrolled group (*n* = 18), the study was underpowered to draw definitive conclusions regarding safety. In our cohort, many uncontrolled patients had received adjunctive therapies such as Lugol’s iodine and corticosteroids to improve perioperative stability. Thus, while their thyroid hormone levels exceeded reference ranges, they may have achieved partial clinical control. Moreover, the uncontrolled group mainly consisted of patients with urgent surgical indications, medication intolerance, or refractory symptoms. This context highlights that surgery in a biochemically uncontrolled state was not undertaken routinely, but rather under specific clinical circumstances and after multidisciplinary evaluation.

Thyroidectomy is a well-established therapeutic option for the definitive treatment of hyperthyroidism. Our observations add to the ongoing debate on whether surgery should proceed in patients who remain biochemically uncontrolled, as the safety of this approach remains controversial because of the potential risk of thyroid storm and other hemodynamic instabilities.

Achieving a euthyroid state prior to thyroid surgery is generally recommended to minimize intraoperative and postoperative complications [11, 12]. However, several studies have reported that thyroidectomy may still be safely performed in patients who remain biochemically hyperthyroid at the time of surgery [3, 4, 6, 16–19]. In addition, some evidence suggests that early surgical intervention may improve the overall health status and expedite the resolution of symptoms in patients with hyperthyroidism [20]. Nevertheless, the potential risks associated with operating on patients in a hyperthyroid state must be carefully considered.

Thyroidectomy is considered an effective treatment option for thyrotoxicosis secondary to amiodarone use. In a multidisciplinary study involving patients with amiodarone-induced thyrotoxicosis who underwent total thyroidectomy under general anesthesia without achieving a preoperative euthyroid state, no significant intraoperative or postoperative complications were observed, and no cases of thyroid storm were reported. Based on these findings, the authors recommended early surgical intervention without delaying for medical stabilization [21]. Similarly, a pediatric study evaluating total thyroidectomy in children with Graves’ disease found that intraoperative hyperthyroidism did not increase the risk of surgical complications although it was associated with elevated heart rate. The authors concluded that the presence of biochemical hyperthyroidism should not delay surgical treatment [22]. It should be noted that our study did not include patients with amiodarone-induced thyrotoxicosis or individuals under 18 years of age.

One study reported no cases of thyroid storm in patients who underwent total thyroidectomy for hyperthyroidism, even in the absence of preoperative Lugol solution or potassium iodide. Moreover, the overall complication rates were low, regardless of the underlying etiology, including Graves’ disease and toxic multinodular goiter [23]. In another study of 227 patients who underwent total thyroidectomy, approximately one-third were in the hyperthyroid phase at the time of surgery. Subgroup analysis focusing on patients with Graves’ disease demonstrated no significant differences in postoperative complication rates—such as transient hypocalcemia, transient hoarseness, and hematomas requiring surgical evacuation—between euthyroid and hyperthyroid individuals. Notably, all procedures in this study were performed by high-volume endocrine surgeons, a factor emphasized as critical to achieving safe outcomes [24].

A study comparing patients who were biochemically hyperthyroid with those who were euthyroid at the time of total thyroidectomy monitored intraoperative parameters associated with thyrotoxicosis, such as heart rate > 100 bpm, systolic blood pressure > 180 or < 60 mmHg, and temperature > 38 °C. No significant differences were found between the groups in terms of these intraoperative findings, except for an increased need for beta-blockers among patients with hyperthyroidism. The authors concluded that thyroidectomy can be safely performed in patients with mild to moderate biochemical hyperthyroidism, provided that the surgical and anesthetic teams are experienced, with no observed differences in mortality, length of postoperative hospital stay, or intraoperative outcomes related to thyrotoxicosis [25]. Consistent with these findings, our study also found no significant differences in complication rates between patients who were biochemically controlled and those who were not at the time of surgery.

A study evaluating the American Thyroid Association (ATA) guidelines reported that while preoperative medical therapy is important for symptom control, patients who remain biochemically hyperthyroid can still achieve favorable surgical outcomes [26]. However, a systematic review encompassing 26 studies concluded that no specific subgroup of patients at increased risk for perioperative thyroid storm could be reliably identified, and thus recommended preoperative treatment whenever possible [27]. Taken together, these findings highlight the ongoing debate regarding the necessity of achieving euthyroid status before thyroidectomy. Our results add to this discussion by supporting individualized, context-specific decision-making—particularly in high-volume centers with multidisciplinary teams experienced in the comprehensive management of patients with hyperthyroidism.

This study has several limitations. The retrospective design introduces the potential for selection and information bias. In addition, the number of patients in the uncontrolled group was relatively small (*n* = 18), which limited the statistical power to detect rare adverse outcomes such as thyroid storm. Because of the small sample size and low number of complications, particularly in the uncontrolled subgroup, a multivariate logistic regression analysis was not feasible without risking model overfitting and unstable estimates. Furthermore, most procedures were performed in a high-volume endocrine surgery unit by experienced surgeons at a tertiary care center; therefore, our findings may not be generalizable to lower-volume or non-specialist settings. Taken together, these limitations highlight the need for cautious interpretation of our results.

## Conclusions

In this retrospective study, thyroidectomy was feasible in a subset of patients who remained biochemically hyperthyroid despite medical therapy, particularly when performed in a high-volume tertiary center. No thyroid storm was observed, and complication rates did not differ significantly from biochemically controlled patients.

Nevertheless, biochemical euthyroidism should remain the standard preoperative target, and surgery in uncontrolled patients should be reserved for carefully selected or urgent cases. Our findings support the feasibility of this approach under specific institutional conditions but do not establish general safety. Larger, prospective multicenter studies are required to validate these results and to better define the risk profile of thyroidectomy in biochemically uncontrolled hyperthyroidism.

## Data Availability

The datasets generated during and/or analysed during the current study are available from the corresponding author on reasonable request.
